# Surface Plasmon Resonance Investigations of Bioselective Element Based on the Recombinant Protein A for Immunoglobulin Detection

**DOI:** 10.1186/s11671-017-1903-5

**Published:** 2017-02-10

**Authors:** A. Bakhmachuk, O. Gorbatiuk, A. Rachkov, B. Dons’koi, R. Khristosenko, I. Ushenin, V. Peshkova, A. Soldatkin

**Affiliations:** 1 Institute of Molecular Biology and Genetics of National Academy of Sciences of Ukraine, 150 Zabolotnogo Str., 03680 Kyiv, Ukraine; 2 Institute of High Technologies, Taras Shevchenko National University of Kyiv, 2, korp.5, Pr. Akademika Hlushkova, Kyiv, 03022 Ukraine; 3 State Institute of Genetic and Regenerative Medicine, NAMS of Ukraine, 57/3, Velyka Vasyl’kivska Str., Kyiv, 03150 Ukraine; 4 Institute of Pediatrics, Obstetrics and Gynecology, NAMS of Ukraine, 8, Maiborody Str., Kyiv, 04050 Ukraine; 5 V.E. Lashkaryov Institute of Semiconductor Physics, NAS of Ukraine, 41, Prospect Nauki, Kyiv, 03028 Ukraine

**Keywords:** Immunoglobulin, Recombinant Staphylococcal protein A, Surface plasmon resonance, Protein immobilization, Affinity biosensor

## Abstract

The developed surface plasmon resonance (SPR) biosensor based on the recombinant Staphylococcal protein A with an additional cysteine residue (SPA-Cys) used as a biorecognition component showed a good selectivity and sensitivity for the immunoglobulin detection. The developed biosensor with SPA-Cys-based bioselective element can also be used as a first step of immunosensor creation. The successful immobilization of SPA-Cys on the nanolayer gold sensor surface of the SPR spectrometer was performed. The efficiency of blocking nonspecific sorption sites on the sensor surface with milk proteins, gelatin, BSA, and HSA was studied, and a rather high efficiency of using gelatin was confirmed. The SPR biosensor selectively interacted with IgG and did not interact with the control proteins. The linear dependence of the sensor response on the IgG concentration in the range from 2 to 10 μg/ml was shown. Using the calibration curve, the IgG concentration was measured in the model samples. The determined concentrations are in good agreement (*r* ^2^ = 0.97) with the given concentration of IgG.

## Background

Determination of the serum level of immunoglobulins is very important for the diagnosis of autoimmune and immunodeficiency diseases, for example, X-linked agammaglobulinemia and common variable immunodeficiency syndrome [1, 2]. Therefore, a lot of methods for IgG detection were developed: electrophoresis [3], radial immunodiffusion [4, 5], agglutination test, enzyme-linked immunosorbent assay (ELISA) [6–8], and immunoblotting [8].

Compared with the existing conventional analytical approaches, the biosensors have several advantages: they provide easy, fast, accurate, highly sensitive, specific, and cheap procedure of measurement. A biosensor is a self-contained device consisting of two functional parts: a bioselective element that specifically reacts with analyte and a physical transducer, which transforms the information from a biorecognition part into an electrical or an optical signal suitable for further processing and characterization [9–13].

The real-time label-free method based on the surface plasmon resonance (SPR) spectrometry for the biosensor development seems to be especially promising and attractive, because it can overcome some serious shortcomings of the methods based on the use of molecular labels (time- and cost-consuming preparation of labeled components, possible influence of labels on interacting biomolecules, multi-step detection protocols, etc.) [14]. The immobilization of biomacromolecules on the 50-nm-thick gold sensor surface and interactions of the immobilized components with their molecule partners change the refractive index of a narrow layer (~200 nm) of media adjacent to the sensor surface. The SPR spectrometer registers these changes [15]. Unlike the well-known, but extremely expensive, bench-top “Biacore” SPR spectrometers, the “Plasmon” SPR spectrometers developed at the V.E. Lashkaryov Institute of Semiconductor Physics of National Academy of Sciences of Ukraine are small, rather simple in operation, and much cheaper devices [16, 17].

One of the best candidates for creation of the bioselective element of the SPR biosensor, which can detect the amount of IgG in the sample, is immunoglobulin-binding Staphylococcal protein A (SPA). It also can be used to form an intermediate layer at creation of immunosensors, because SPA selectively binds the Fc fragment of the antibody leaving the Fab fragment available for antigen detection. The SPA molecule structure includes a signaling sequence [18], an IgG-binding region consisting of five highly homologous domains, and a C-terminal anchoring part, which attaches the protein to the bacterial cell wall [19, 20]. The SPA molecules are highly resistant to denaturing factors: they are thermostable and resistant to a wide range of pH (1–12) and to trypsin cleavage [21].

The immunoglobulin-binding region of SPA does not contain cysteine residues [22, 23], so the immobilization of SPA on the gold sensor surface is possible only by physical sorption, which is not always reliable. To overcome this limitation, a specific attachment site is selectively introduced into a nonessential part of the recombinant protein A using a genetic engineering approach. It was shown that the introduction of a cysteine residue in the recombinant protein A increases the immobilization level of SPA via a strong interaction of the thiol group with the gold sensor surface. Moreover, this variant of immobilization of SPA improves the IgG-binding activity of SPA as well as further antigen-binding activity of antibody molecules immobilized through SPA [24]. In our previous work, the original plasmid *pET24-SPA-6HisCys* was constructed, the recombinant protein A contained all five IgG-binding domains, 6His-tag and C-terminal cysteine residue (SPA-Cys) was obtained, and its immobilization on the gold sensor surface was demonstrated [25].

The aim of this work was to investigate the formation of bioselective element based on recombinant Staphylococcal protein A with additional cysteine residue (SPA-Cys) for immunoglobulin detection using the SPR spectrometer “Plasmon.”

## Methods

NaCl, KH_2_PO_4_, bovine serum albumin (BSA), and human IgG were purchased from “Sigma” (USA); Na_2_HPO_4_, from “Applichem” (Germany); milk proteins (skim milk powder), from “Fluka” (Switzerland); human serum albumin, from “Biofarma” (Ukraine); and other reagents and solvents were obtained from “UkrOrgSyntez” (Ukraine). Sodium phosphate saline buffer solution (PBS), which includes 10 mM Na_2_HPO_4_, 1.76 mM KH_2_PO_4_, 137 mM NaCl, 2.7 mM KCl, and pH 7.4, was used as working buffer.

Synthesis and purification of recombinant Staphylococcal protein A with specially introduced C-terminal cysteine residue (SPA-Cys) were described in [25]. The homogeneity of the proteins used was checked by electrophoresis in 13% polyacrylamide gel under denaturing conditions.

The SPR spectrometer “Plasmon SPR-4m” was used to study the protein-protein interactions (the device and corresponding software have been developed at the V.E. Lashkaryov Institute of Semiconductor Physics, NAS of Ukraine). The optical phenomenon of SPR in Kretschmann configuration is used in this computer-controlled optoelectronic spectrometer. A thin layer (50 nm) of gold, which was deposited on a glass plate, is used as a sensitive element of the SPR spectrometer. Just before the experiment, the plate gold surface was purified by incubation in a mixture of “piranha” (a mixture of 30% H_2_O_2_ and concentrated H_2_SO_4_ in 1:3 ratio) for 2 min. Then the plate was repeatedly washed with water and dried in air. After that, it was mounted on the device prism using the immersion liquid with the same refraction index as the prism and the glass plate. The gold surface serves as a bottom of a flow measuring cell (~20 μl). A silicone rubber ring serves as the side walls. A Plexiglas cover contains input and output pipes, which the buffer solution and investigated samples pass through. The flow rate of liquid (usually 40 μl/min) is controlled by the peristaltic pump “Ismatec” (Switzerland).

All SPR experiments were performed at room temperature. At the beginning of the experiment, the measuring cell was washed with the working buffer solution (PBS), to obtain a stable signal of the device—the baseline. For immobilization, 120 μl of 1 μM solution of SPA-Cys in PBS was injected into the measuring cell. After the 25-min incubation of the sample, the excess of unbound proteins was removed by a flow of the working buffer solution.

To prevent nonspecific adsorption on the surface sites, which are left uncovered with immobilized proteins, the sensor surface was passivated with other proteins such as gelatin, milk proteins, BSA, or HSA. For this purpose, the consecutive injections of 120 μl of 0.2 mg/ml protein solution in PBS into the measuring cell were made followed by the incubation for 20 min and washing the surface with PBS until stabilization of the sensor signal [26].

Then 120 μl of the solutions of various IgG concentrations in PBS was injected into the measuring cell, incubated for 10 min with subsequent washing of the cell with PBS until stabilization of the sensor signal.

For regeneration of the bioselective element (a destruction of bonds between the immobilized protein A and IgG as well as a removal of the latter), 120 μl of 40 mM citrate buffer (pH 2.5) was injected into the measuring cell, then the cell was washed with PBS until stabilization of the sensor signal [27].

## Results and Discussion

**Fig. 1:**
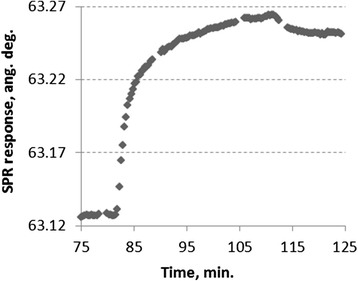
SPR sensogram representing the immobilization of 1 μM SPA-Cys on the gold sensor surface of SPR spectrometer “Plasmon-4m”

For the SPA-Cys immobilization on the gold sensor surface of the SPR spectrometer “Plasmon-4m,” a sample of the purified recombinant Staphylococcal protein A was injected into the measuring flow cell and incubated. It led to a significant increase in the sensor signal (Fig. 1). After washing of the flow cell with PBS, a slight decrease in the sensor signal was observed. It means that most of the SPA-Cys molecules were tightly immobilized on the gold sensor surface. The difference between the signals before the injection of 120 μl of 1 μM SPA-Cys and after washing with PBS was almost 0.12 angular degrees. It represents a level of the reliably immobilized SPA-Cys molecules.

The dependence of the immobilization level on the concentration of SPA-Cys was investigated. A linear part of this dependence was observed in the range from 0 to 0.5 μM SPA-Cys, whereas 2 μM SPA-Cys corresponds to the close-to-saturation level of immobilization [26]. According to the conversion factor of the SPR response into the value of surface density of the immobilized protein [28], this value at 2 μM SPA-Cys was 1.1 ± 0.2 ng/mm^2^. Given the molecular weight of SPA-Cys (34.5 kDa), we can calculate that in average, approximately 51 nm^2^ of the sensor surface falls on one molecule of the immobilized SPA-Cys. Therefore, no dense monolayer of proteins is formed on the sensor surface. In this regard, the need of blocking efficiently nonspecific adsorption on the free sites is obvious.

**Fig. 2:**
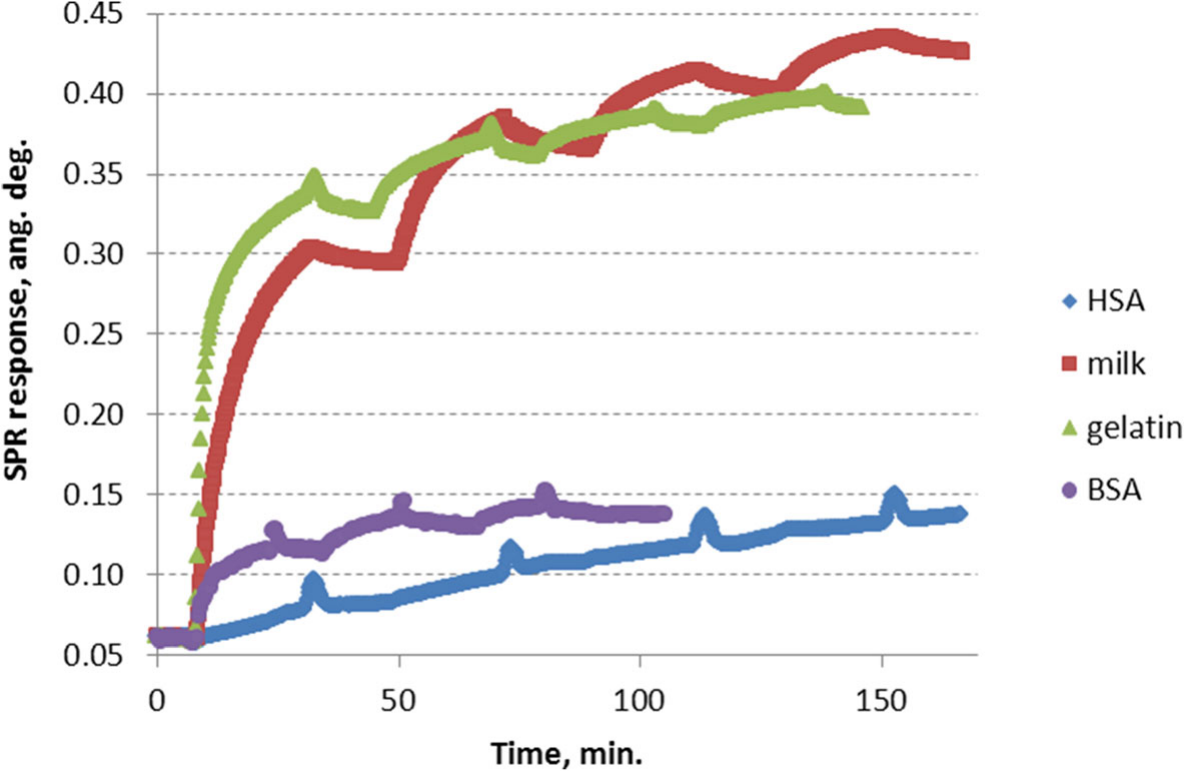
The SPR sensograms representing the process of immobilization of the blocking agents on the gold sensor surface: HSA (*blue*), BSA (*violet*), gelatin (*green*), and milk proteins (*red*)

The attempt to apply BSA as a blocking agent did not give a satisfactory result: the saturation of the sensor response was achieved after three consecutive injections of BSA into the measuring flow cell, and its growth (~0.08 angular degrees) corresponds to ~0.8 ng/mm^2^ only (Fig. 2, violet). Even taking into consideration a bigger size of BSA molecules, the average value 41 nm^2^ of the sensor surface per each immobilized protein molecule indicates on a quite large area of free sensor surface [26].

Application of HSA as a blocking agent gave even worse results. After four consecutive injections of HSA into the measuring flow cell, the growth of the sensor response corresponded to ~0.7 ng/mm^2^ only (~0.07 angular degrees) (Fig. 2, blue).

In contrast to albumins, milk proteins (solution of the skim milk powder) demonstrated a high efficiency when being applied for the blocking procedure (Fig. 2, red) [26]. However, after few days of exploitation with regeneration of the sensor surface, the injections of, for example, 40 μg/ml IgG resulted in the SPR response, which was much larger than the usual one. It can be explained by the assumption that the regeneration buffer solution washes out some molecules of the blocking layer. As a result, an additional portion of IgG molecules could bind nonspecifically to the bare gold surface. The injection of an additional portion of milk protein solution led the SPR response on IgG to the return to its usual level.

Therefore, in the present work, the efficiency of blocking the nonspecific adsorption sites by gelatin was investigated. The first injection of gelatin into the measuring flow cell caused a very big (~0.3 angular degrees) sensor response. Washing the measuring cell with PBS led to a small signal reduction (reflecting a relatively small portion of weakly adsorbed molecules compared with strongly immobilized ones). Each following injections gave a gradually decreasing increment of the sensor response, and four consecutive injections of gelatin were necessary to get the saturation of the sensor response (~0.33 angular degrees) (Fig. 2, green). It corresponds to the protein surface density of ~3.3 ± 0.1 ng/mm^2^. This value indicates on a quite dense protein monolayer.

**Fig. 3:**
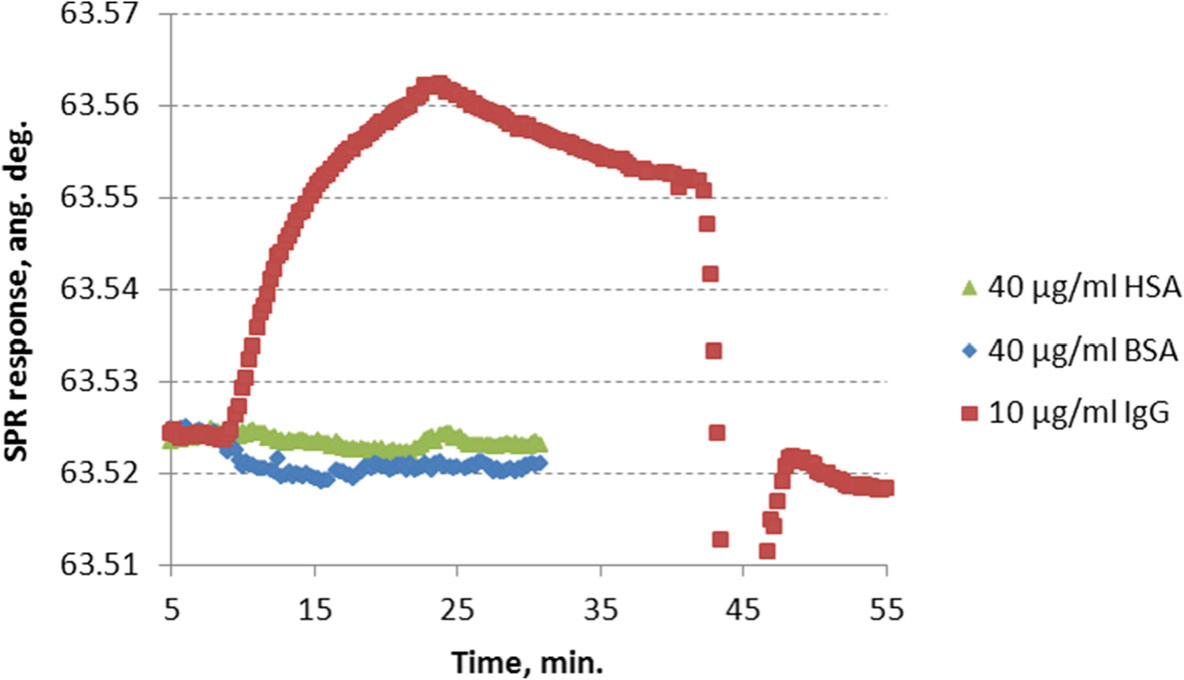
SPR sensograms representing the interactions of the bioselective element based on SPA-Cys immobilized on a gold sensor surface with 10 μg/ml IgG (*red*), 40 μg/ml HSA (*green*), and 40 μg/ml BSA (*blue*)

To check whether the immobilized molecules of SPA-Cys retain their immunoglobulin-binding properties, human IgG was injected into the measuring flow cell. The sensogram (Fig. 3) shows that the injection of 10 μg/ml IgG causes a significant sensor response without essential decrease during prolonged PBS wash. Obviously, the molecules of SPA-Cys immobilized on the gold sensor surface demonstrated their high immunoglobulin-binding activity.

For comparison, the injections of other proteins (40 μg/ml BSA or 40 μg/ml HSA) do not cause noticeable changes in the sensor response (Fig. 3). HSA is present in the blood samples, and it is important to know that it does not interact with SPA-Cys or the blocking layer. Thus, the created bioselective element of the SPR biosensor based on the recombinant protein SPA-Cys and passivated by gelatin demonstrated high selectivity of the sensor response.

For further use of the prepared bioselective element, an efficient regeneration procedure should be developed. For this purpose, the use of various solvents or reagents that change the pH and/or ionic strength of the solution inside the measuring flow cell acting on a charge of interacting molecules and thus their tertiary structure was described [29]. In this work, after treating the bioselective element with bound IgG with 40 mM sodium citrate buffer (pH 2.5) [27], the level of sensor signal almost backs the values that preceded the IgG injection (Fig. 3). It shows quite effective disruption of the links between the immobilized SPA-Cys and IgG and removal of the latter. The subsequent injections of new IgG samples showed that such regeneration procedure has not essentially affected the level of immunoglobulin-binding activity of the immobilized SPA-Cys. Thus, the re-use of the immunosensor bioselective element formed on the basis of SPA-Cys is possible.

**Fig. 4:**
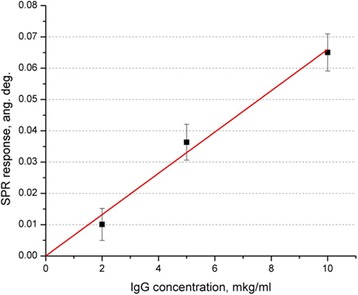
The dependence of the sensor response on the concentration of IgG

The dependence of the sensor response on the IgG concentration was received by measuring the IgG samples (from 2 to 10 μg/ml) after the SPA-Cys immobilization at 1 μM concentration (surface density ~0.6 ng/mm^2^) and blocking nonspecific sorption sites by gelatin (Fig. 4).

**Fig. 5:**
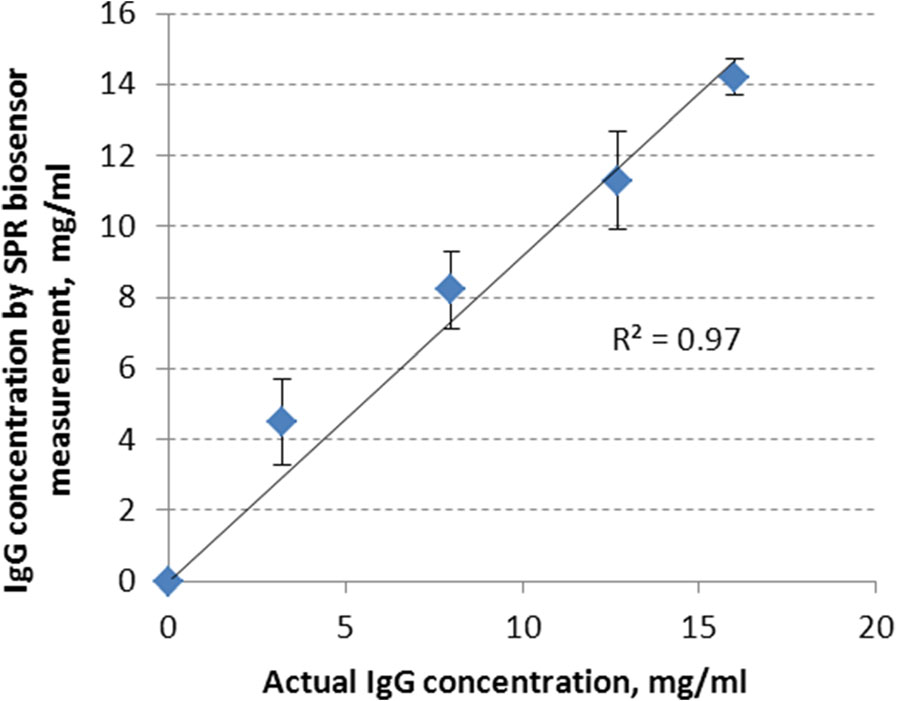
The correlation between the IgG concentrations measured by the SPR biosensor based on SPA-Cys and the actual IgG concentrations

To test the developed biosensor, five solutions with different given IgG concentrations in PBS were measured. Figure 5 shows that the experimentally determined results are in good agreement with the actual concentrations of IgG in the model samples.

## Conclusions

The successful immobilization of recombinant protein A from *Staphylococcus aureus* with the C-terminal cysteine residue (SPA-Cys) on the nanolayer gold sensor surface of the SPR spectrometer while preserving its high immunoglobulin-binding activity and selectivity of the sensor response has been performed. The suitability of SPA-Cys as a bioselective component for the creation of the biosensor for the IgG detection has been shown. The efficiency of blocking nonspecific adsorption sites on the sensor surface with milk proteins, gelatin, BSA, and HSA was studied, and a rather high blocking ability of gelatin was revealed. The linear dependence of the sensor response on the IgG concentration (from 2 to 10 μg/ml) allows determining IgG concentration in the model samples by using a calibration curve. The biosensor developed is a perspective for the evaluation of the immune status. For this purpose, further investigations should be directed on the study of possible influence of other components of the serum on the biosensor response and a possibility of its usage as an express method for the determination of the IgG level in the real biological samples. Moreover, we believe that our study has shown an ability of SPA-Cys to perform a function of an intermediate layer in the bioselective element for the creation of the immune biosensor.

## Abbreviations

BSA: Bovine serum albumin
HSA: Human serum albumin
PBS: Sodium phosphate saline buffer solution
SPA: Staphylococcal protein A
SPA-Cys: Recombinant Staphylococcal protein A with an additional cysteine residue
SPR: Surface plasmon resonance
